# Cerebral Autosomal Dominant Arteriopathy With Subcortical Infarcts and Leukoencephalopathy Presenting During the Postpartum Period as Postpartum Depression and Postpartum Psychosis

**DOI:** 10.7759/cureus.39099

**Published:** 2023-05-16

**Authors:** Mansoor Zafar, Meera Gajre, Aparna Balagopal, Syed Ashhar Naqvi, Darius S Khalesi, Rusiru Premathilaka, Ariful Islam, Stefano Berliti, Garabedyan Hovagim, Kadir Hacikurt

**Affiliations:** 1 Gastroenterology/General Internal Medicine, Royal Sussex County Hospital, University Hospitals Sussex National Health Service (NHS) Foundation Trust, Brighton, GBR; 2 General Internal Medicine, Conquest Hospital, St. Leonards-on-Sea, GBR; 3 Internal Medicine, Conquest Hospital, St. Leonards-on-Sea, GBR; 4 Emergency Medicine, Conquest Hospital, St. Leonards-on-Sea, GBR; 5 Acute Medicine, Conquest Hospital, St. Leonards-on-Sea, GBR; 6 Neurology, Conquest Hospital, St. Leonards-on-Sea, GBR; 7 Radiology, Conquest Hospital, St. Leonards-on-Sea, GBR

**Keywords:** cadasil, mri flair sequences, mri t2-weighted, granular osmiophilic material (gom), chromosome 19, notch 3 mutation

## Abstract

Cerebral autosomal dominant arteriopathy with subcortical infarcts and leukoencephalopathy (CADASIL) is a rare inherited disease that presents with neurologic manifestations such as stroke, psychiatric disturbances, migraine, and cognitive decline. We report a case of a previously well 27-year-old lady presenting with new onset confusion four weeks postpartum. On examination, there was right-sided weakness and tremors. A thorough history revealed existing diagnoses of CADASIL in 1^st^ and 2^nd^-degree relatives. The diagnosis in this patient was confirmed by MRI of the brain and genetic testing for *NOTCH 3* mutation. The patient was admitted to the stroke ward, treated with a single antiplatelet agent for stroke, and supported by speech and language therapy. There was a significant symptomatic improvement in her speech at the time of discharge. The mainstay of treatment for CADASIL remains symptomatic at this stage. This case report shows that the first presentation of CADASIL can mimic postpartum psychiatric disorders in a puerperal woman.

## Introduction

Cerebral autosomal dominant arteriopathy with subcortical infarcts and leukoencephalopathy (CADASIL), is a rare hereditary condition, associated with mutations in the *NOTCH 3* gene on chromosome 19 [1]. It has been reported as one of the most common types of hereditary stroke affecting the small blood vessels in the cerebral white matter [2]. It is also distinctly characterised by the accumulation of granular osmiophilic material within the cerebral vasculature [3].

Previous studies have reported a prevalence of mutation carriers between 0.8 to 5 per 100,000 individuals [4] while Moreton et al. reported a prevalence to be at least 4.6 per 100,000 adults [5]. Several variants of the *NOTCH3* gene have been identified. Rutten et al. has reported that patients with epidermal growth-factor-like repeat (EGFr) domain 1-6 pathogenic variant of the *NOTCH3* gene have a 12-year earlier onset of stroke than those patients with an EGFr 7-34 pathogenic variant [6]. Also reported is lower survival. Additionally, they have reported among those diagnosed with CADASIL, 70% of CADASIL cases have an EGFr 1-6 pathogenic variants. The EGFr 7-34 pathogenic variants of the *NOTCH3* gene are reported to be associated with a later onset of stroke and longer survival presenting as milder clinical variants that have been found to be more prevalent in people of Asiatic descent [6].

The four most commonly reported presentations of CADASIL are; recurrent ischemic strokes, psychiatric disturbances, migraine with aura, and cognitive decline [7,8]. Of these, the most common is transient ischaemic attacks (TIAs) and infarctions (60-80% of patients) [8]. The most frequent presentation (in 60- 80% of patients) reported is with Cerebral transient ischemic attacks and infarctions [8]. The reported age of onset of CADASIL ranges between 20 to 70 years [8].

## Case presentation

A 27-year-old female presented to the emergency department (ED) of a district general hospital, with new confusion four weeks post-partum. Her partner reported she had developed difficulty with using her mobile phone initially. Then she appeared disorientated, drowsy, and had generalised apathy. She was unable to recognise her family and her communication was limited to monosyllabic answers to questions with ‘yes’, ‘no’, or ‘I don’t know’. Her observations including vital signs and routine blood tests were normal and she was referred from the ED to the psychiatric team for management of postpartum depression and/or psychosis. When reviewed by psychiatry, a second opinion from the medical team was requested to rule out a medical cause for her confusion.

**Figure 1:**
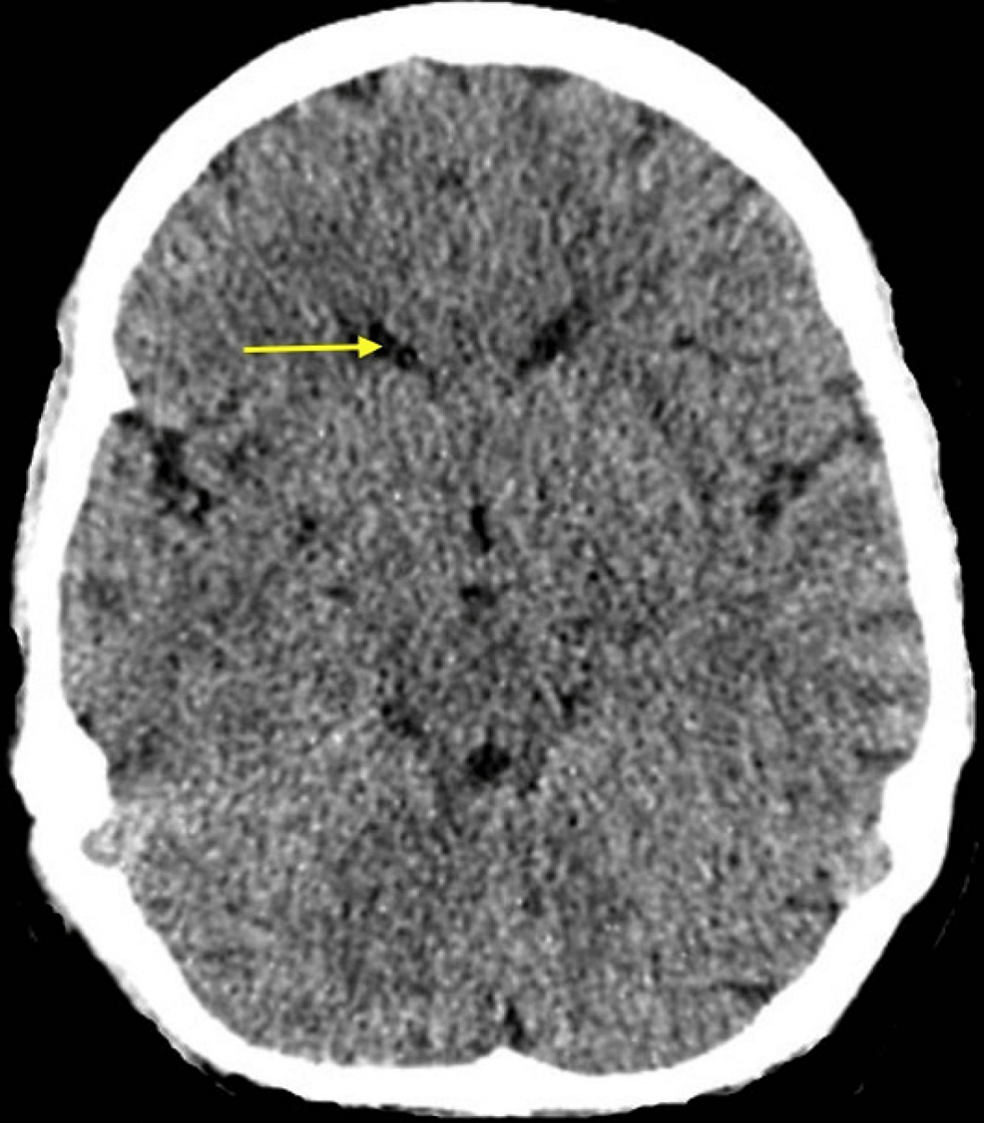
Computed tomography (CT) scan of the head Normal appearances of the brain parenchyma with preserved grey-white matter differentiation. No hyperdensity is identified on the venous sinuses to suggest underlying venous sinus thrombosis. Normal ventricular configuration (yellow arrow). No midline shift or hydrocephalus. No destructive skull lesions.

The medical team noted she had a normal pregnancy with a caesarean section delivery. She had no past medical history and was taking no regular medications. There was no history of fevers before admission however, she had a toothache which was being managed with oral antibiotics from the dentist for the last two weeks. On examination, the patient looked drowsy. Observations showed a respiratory rate of 17/minute, heart rate of 96/minute, temperature of 38.8 degrees Celsius, blood pressure of 123/86 mmHg, and oxygen saturation of 97% on air. The systemic review revealed normal heart sounds and breathing on auscultation, a soft and non-tender abdomen, a healing caesarean section scar with no organomegaly and no lower limb oedema. Her Glasgow coma scale score (GCS) was 14/15 due to confusion. Neurological exam revealed an inability to follow commands with her right hand. Muscle power assessment was 5/5 in all 4 limbs with right-sided intension tremor present with no nystagmus. An urgent computerised tomography (CT) scan of the head showed no bleeding or any ischaemic changes in the brain (Figure 1).

**Figure 2:**
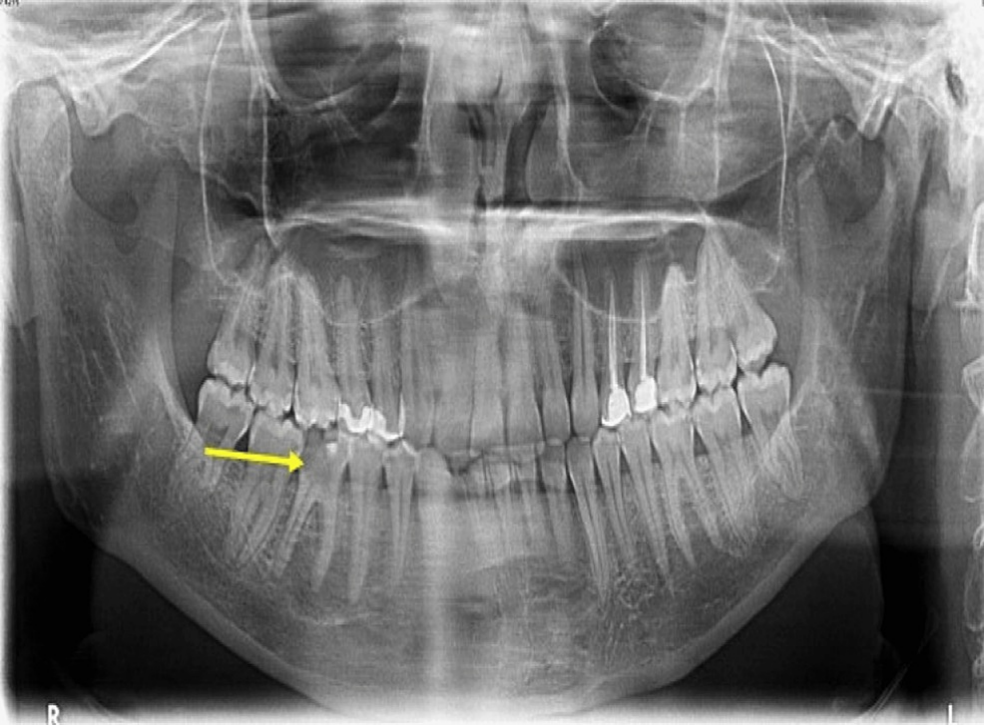
Orthopantomogram image Dental caries were noted on the lower right first molar tooth, but no obvious lucency to the surrounding bone to indicate a bony infection.

Given her presentation with right sided weakness and fever, an initial differential diagnosis of stroke or cerebral-venous thrombosis and/or meningo-encephalitis was made. She was started on intravenous acyclovir, ceftriaxone, and high-dose aspirin of 300 mg once a day and admitted to the stroke ward. Additionally based on the history of a tooth ache an X-ray orthopantomogram was also requested. This showed multiple fillings and dental caries in the right first lower molar tooth, but no obvious lucency to the surrounding bone to indicate any bony infection. The images were reviewed by the maxillofacial consultant, who advised no abscess seen and for dental review once patient was stable(Figure 2).

**Figure 3:**
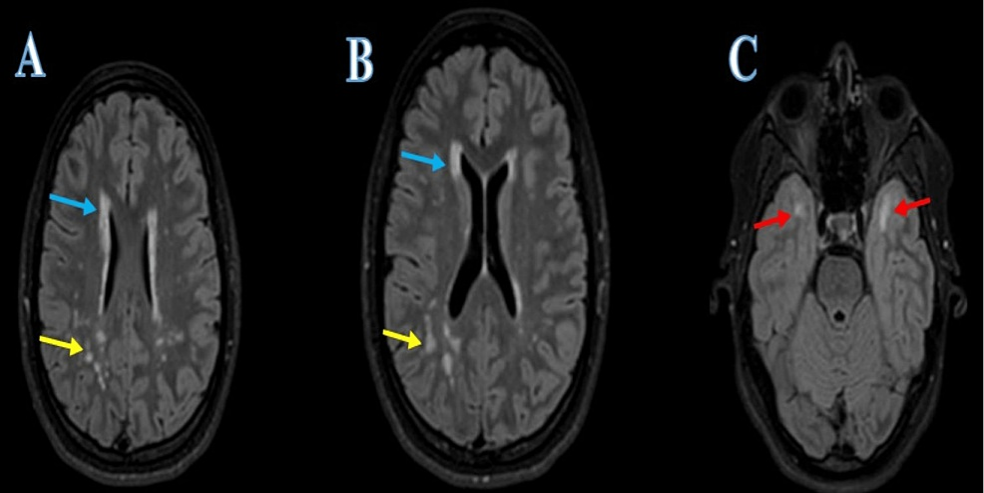
MRI image of patient's head A. Extensive white matter abnormality with multiple foci of increased fluid-attenuated inversion recovery (FLAIR) and T2 signal evident within the periventricular white matter and subcortical white matter in the frontal (blue arrows) band parietal lobes (yellow arrows). B. Confluent areas of the abnormal signal were noted in the periventricular right frontal lobe (blue arrows) and parieto-occipital lobe (yellow arrows). C. FLAIR hyperintensity is evident in the subcortical white matter of the anterior temporal lobes (red arrows), a typical location of CADASIL.

To check for meningo-encephalitis a lumbar puncture was performed and cerebrospinal fluid (CSF) analysis showed no evidence of infection. The medical team at this stage queried for cerebral autosomal dominant arteriopathy with subcortical infarcts and leukoencephalopathy (CADASIL) and requested magnetic resonance imaging (MRI) of head along with magnetic resonance venogram (MRV) which confirmed extensive white matter signal abnormality with a strong suspicion for CADASIL (Figure 3).

Following the test results, the medical team contacted the patient’s family to get a further history of any ailments in the family and/or inherited disorders based on new onset confusion with vague unilateral right sided intention tremors and cerebellar signs along with MRI findings. The patient’s family advised that the patient’s father and one of her paternal uncles do suffer from CADASIL and under care at a tertiary hospital.

**Figure 4:**
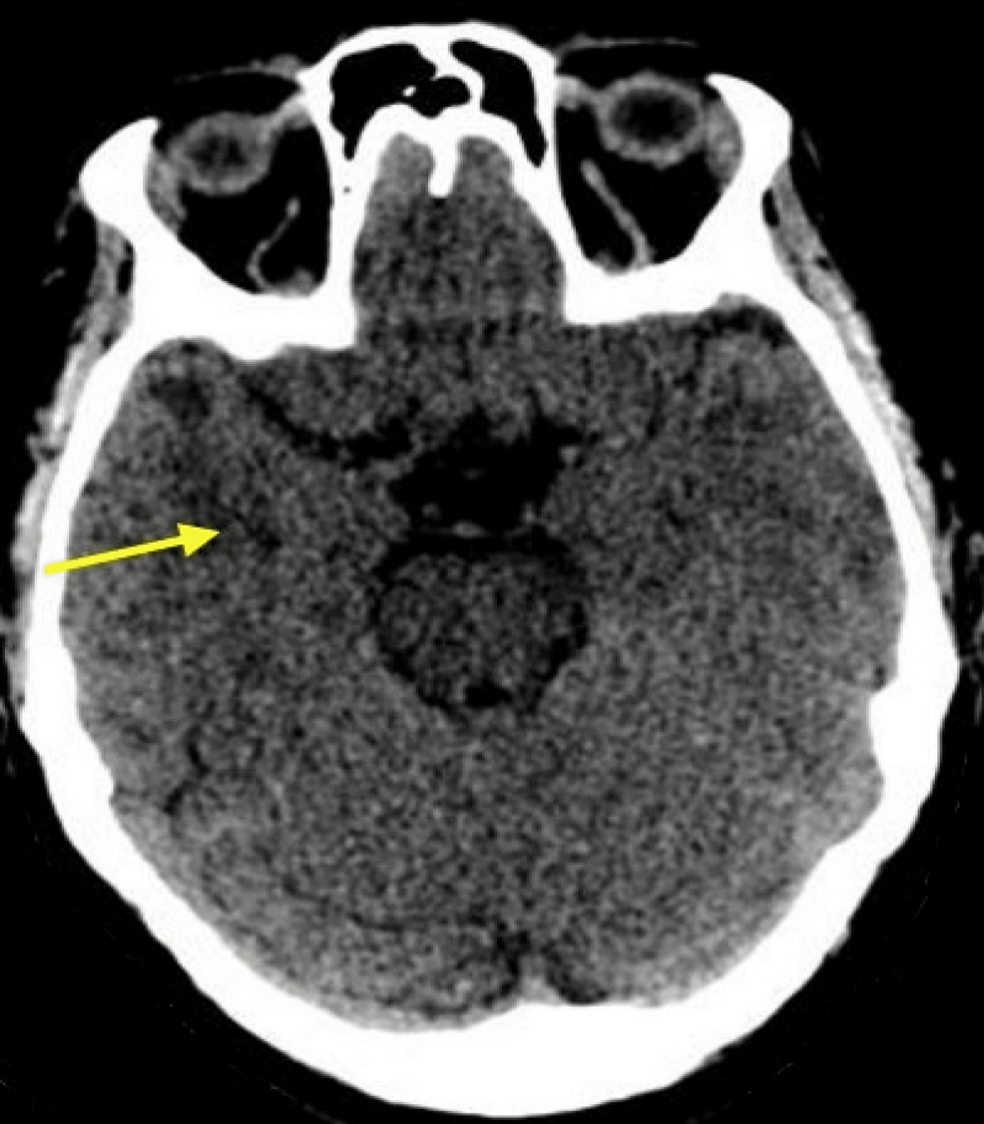
Repeat CT head image after seven days Bilateral anterior temporal lobe infarcts with extensive periventricular and deep white matter low attenuation change throughout the cerebrum with a right-side predominance demonstrated and is more evident than on the previous CT head (yellow arrow).

She continued to be treated with aspirin 300 mg once a day (OD) while an inpatient under the stroke team. On the seventh day of admission, she developed status epilepticus for which she was intubated and was transferred to intensive care unit (ITU). Here she was started on intravenous levetiracetam. A repeat CT head showed extensive white matter changes that had progressed since the previous CT head seven days ago (Figure 4).

The patient also had in-patient echocardiogram that showed normal left ventricular size with good systolic function no valvular abnormality and ejection fraction of 60-65%. Her case was discussed with the neurology team who advised to continue with levetiracetam for seizures and request electroencephalogram (EEG) and battery of blood tests (Table 1).

**Table 1:** Blood test results Source: Laboratory at Conquest Hospital, East Sussex Healthcare NHS Trust. IgG: Immunoglobulin G; IgM: Immunoglobulin M; HbA1C: Haemoglobin A1C; CSF: Cerebrospinal fluid; PCR: Polymerase chain reaction

Parameter	Unit of measurement	Normal range	Patient’s blood test results
Anti DNA antibody	IU/ml	0- 10	1
Anti myeloperoxidase antibody	IU/ml	0-3.4	< 0.2
Anti proteinase 3	IU/ml	0-1.9	< 0.2
N-methyl-D-aspartate	-	negative	negative
HIV antibody/antigen	-	negative	negative
Syphilis IgG/IgM screen test	-	negative	negative
HbA1c	mmol/mol	21-41	29
Rheumatoid factor	IU/ml	0-14	<10
Erythrocyte sedimentation rate (ESR)	mm/h	3-15	8
Contactin-2-assisted protein antibody	-	negative	negative
IgG cardiolipin	GPL U/ml	0-40	1.5
IgM cardiolipin	MPL U/ml	0-40	4.1
Leucine-rich Glioma Inactive antibody	-	negative	negative
CSF viral PCR	-	negative	negative
Plasma glucose	mmol/l	2.5-11	4.6
CSF glucose	mmol/l	Usually 60-80% of Plasma glucose	3.2
CSF protein	g/l	0.15-0.45	0.33
CSF oligoclonal bands	-	negative	negative
Serum thyroid stimulating hormone (TSH)	miu/L	0.27-4.2	0.43
Serum Vitamin B12	ng/L	197-771	279
Serum folate	ug/L	2.4-17.5	3.3

**Figure 5:**
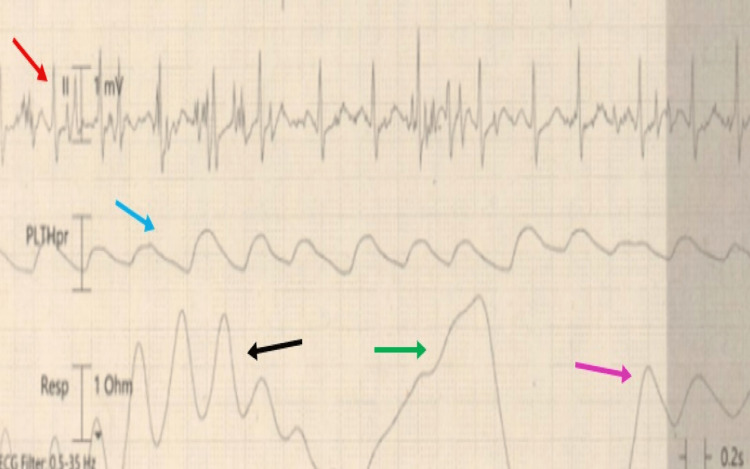
Electroencephalogram Generalised excess of slow activities, which often appears as semi-rhythmic non-evolving runs of high amplitude 1-2 Hz activity (green arrow), maximal over the anterior regions of 4-7 Hz (pink arrow) and with superimposed mixed faster rhythms of 13-15 Hz (black arrow). Indicating significant asymmetric global cortical dysfunction which correlates with the reduced movements noted on the right with no epileptiform or sub-clinical seizure activity and no features suggestive of non-convulsive status. Simultaneous breathing pattern (blue arrow) and ECG rhythm (red arrow).

The EEG showed no epileptiform activity but significant asymmetric global cortical dysfunction which correlated with the reduced movements noted on the right side (Figure 5).

The patient was successfully extubated forty-eight hours later and transferred back to the stroke ward. Repeat MRI head showed features of CADASIL with stable appearances. The patient was regularly reviewed by the speech and language (SALT) team on the ward to help improve the speech and communication. On day fifteen, the patient was switched to clopidogrel 75 mg OD and her first lower right molar tooth was extracted. A referral was made to the tertiary centre for genetic testing that confirmed CADASIL. Her speech improved and she began to speak in full sentences. She did at times still struggle with formulating answers to questions. She was deemed medically stable for discharge with outpatient follow-up by the neurology team and community SALT teams.

## Discussion

The most common symptoms reported by Roine et al. in their study of mothers with CADASIL during gestation and puerperium with R133C *NOTCH3 *pathogenic mutation are hemi-paresthesia (76%), hemiparesis (36%), aphasia (65%), and visual disorders (47%) [9].

MRI brain is considered a very useful method to diagnose CADASIL. The white-matter hyperintensity on T2-weighted or the fluid-attenuated inversion recovery (FLAIR) sequences especially involving the temporal lobes are strongly suggestive of CADASIL [10,11]. Other reported MRI findings are; subcortical lacunar infarcts [12], dilated perivascular spaces, commonly noticed around the basal ganglia, microhemorrhages, and brain atrophy [13]. Genetic testing for *NOTCH3* mutation remains the gold standard for diagnosis [14]. Skin biopsy for granular osmiophilic material (GOM) deposition using electron microscopic examination remains another useful way to diagnose CADASIL [14] although it is a possibility that at times skin biopsy may be negative [15].

Various novel approaches toward treatment have been proposed. These include cysteine corrective exon skipping [16], immunotherapy [17], and combined subcutaneous administration of stem cell factor (SCF) and granulocyte colony-stimulating factor (G-CSF) in a mouse model of CADASIL [18].

The current strategy for management is symptomatic treatment. This may include for acute stroke presentation with the administration of intravenous tissue plasminogen activator (tPA) if criteria are met with an understanding that endovascular recanalization would not be useful as CADASIL is a small vessel disease [19]. Aspirin in 75- 300 mg doses followed by clopidogrel can be used in acute stroke outside the thrombolysis window as in our patient [19]. It can also be used for secondary prevention of stroke although the specific benefit remains to be assessed [20]. Acute migraine attacks are reported to respond to paracetamol and antiemetics [21] with avoidance to use of amitriptyline, topiramate, and beta blockers as these may increase cognitive decline [22]. Triptans should be avoided due to their vasoconstrictive effect [23]. Lastly, quetiapine, flupentixol, sodium valproate, and risperidone, have all been reported to be useful in the management of psychiatric symptoms [24]. Caution should be used with suggested caution towards the use of selective serotonin reuptake inhibitors (SSRIs) due to their association with increased risk of ischemic and hemorrhagic stroke [25]. 

We present a unique case of a patient whose initial symptoms suggested postpartum depression and/or psychosis. It was through a thorough approach to clinical examination and history-taking that led to an impression of CADASIL. This was later diagnosed with an MRI head scan and confirmed with genetic testing to be an inherited disorder with an autosomal dominant trait. Last, but never the least, it would be interesting to see more case reports and research on the role of pregnancy or the postpartum period and if the prothrombotic state of pregnancy could be a possible contributing factor for the onset of the symptoms of CADASIL. 

## Conclusions

CADASIL is a rare hereditary condition that can be the cause of stroke in a young person. It can also lead to cognitive symptoms such as confusion. In a young person presenting to the emergency department with confusion and a fever, meningoencephalitis is a common differential to consider. If this patient is postpartum, it is also important to consider cerebral venous sinus thrombosis, postpartum psychosis, and depression. Here we demonstrate that a thorough history and examination revealed a positive family history of CADASIL and a left-sided stroke. With this information investigations such as an MRI head were arranged, and a diagnosis of CADASIL was reached. The first presentation of CADASIL can masquerade as postpartum depression/psychosis in a puerperal woman, as seen in this patient. MRI remains a very useful imaging towards arriving at the diagnosis, although genetic testing remains the cornerstone for confirming the inherited disorder. This case report highlights the need for a thorough evaluation of personal and family history in such presentations. This is a complex condition with the mainstay of treatment being symptom control. There is little information in the literature surrounding presentations in pregnancy. Further research into this field is required to investigate whether the prothrombotic state of pregnancy leads to earlier presentation of CADASIL in those with the *NOTCH 3* gene mutation for the disease.
